# Factors associated with poor medication adherence in patients with Bipolar Disorders

**DOI:** 10.1192/j.eurpsy.2023.466

**Published:** 2023-07-19

**Authors:** H. Jemli, M. Djelassi, Y. Zgueb, R. Zaibi

**Affiliations:** ^1^Psychiatry deparment A, Razi Hospital, Manouba, Tunisia; ^2^Psychiatry deparment A, Razi Hospital, Manouba

## Abstract

**Introduction:**

Treatment adherence in patients living with Bipolar Disorders can influence prognosis and quality of life. It is associated with an increased morbidity and healthcare costs.

**Objectives:**

The aim of our study was to evaluate treatment adherence in a sample of patients living with Bipolar disorders and to determine factors associated with poor adherence.

**Methods:**

We conducted a cross sectional study where we included bipolar patients being treated in psychiatry department A. We developed a survey containing sociodemographic and clinical features. We used the medical adherence rating scale to evaluate treatment adherence.

**Results:**

Our sample consisted of 100 patients with a mean age of 47,5 years old. Sixty seven patients were being treated for bipolar disorder type 1. Medication adherence rate was 64%.

Factors associated with poor medication adherence were being single, an early age of onset, comorbid substance abuse disorder, severe treatment side effects and poor insight.

**Conclusions:**

Poor medication adherence is a major issue for people living with Bipolar Disorders. Clinicians should pay more attention to sociodemographic and clinical factors to predict and enhance treatment adherence.

**Disclosure of Interest:**

None Declared

